# Identification and validation of a two-gene metabolic signature for survival prediction in patients with kidney renal clear cell carcinoma

**DOI:** 10.18632/aging.202636

**Published:** 2021-03-03

**Authors:** Xudong Guo, Zhuolun Sun, Shaobo Jiang, Xunbo Jin, Hanbo Wang

**Affiliations:** 1 Department of Urology, Shandong Provincial Hospital Affiliated to Shandong First Medical University, Jinan 250021, Shandong, China; 2 Department of Urology, Third Affiliated Hospital of Sun Yat-sen University, Guangzhou 510630, Guangdong, China

**Keywords:** kidney renal clear cell carcinoma, metabolism, nomogram, prognostic signature, The Cancer Genome Atlas

## Abstract

Metabolic reprogramming contributes to the high mortality of advanced stage kidney renal clear cell carcinoma (KIRC), the most common renal cancer subtype. This study aimed to identify a metabolism-related gene (MRG) signature to improve survival prediction in KIRC patients. We downloaded RNA sequencing data and corresponding clinical information for KIRC and control samples from The Cancer Genome Atlas database and identified, based on an MRG dataset in the Molecular Signatures Database, 123 MRGs with differential expression in KIRC. Following Cox regression analysis and least absolute shrinkage and selection operator selection, RRM2 and ALDH6A1 were identified as prognosis-related genes and used to construct a prognostic signature with independent prognostic significance. After risk score-based patient separation, stratified survival analysis indicated that high-risk patients showed poorer overall survival than low-risk patients. We then constructed a clinical nomogram that showed a concordance index of 0.774 and good performance based upon calibration curves. Gene set enrichment analysis revealed several metabolic pathways significantly enriched in the target genes. The two-gene metabolic signature identified herein may represent a highly valuable tool for KIRC prognosis prediction, and might also help identify new metabolism-related biomarkers and therapeutic targets for KIRC.

## INTRODUCTION

Renal cell carcinoma (RCC) accounts for 2% to 3% of all adult cancers. Kidney renal clear cell carcinoma (KIRC) is the most common histological subtype, comprising 80% to 90% of RCC cases [1, 2]. The incidence of RCC has risen steadily within the last decades; moreover, RCC exhibits the highest mortality rate among all urologic malignancies, causing ~100,000 deaths worldwide annually [3]. Since clinical manifestations are diverse and lack specificity, up to 30% of KIRC patients are typically diagnosed at advanced stage [4]. Accordingly, and despite improved medical care, the prognosis of metastatic RCC patients is very poor, with a 5-year overall survival (OS) rate of less than 10%. This contrasts starkly with the high 5-year OS rate (up to ~90%) that can be achieved for patients with early-stage KIRC following surgery [5]. Therefore, identifying early diagnostic biomarkers is crucial to define treatment modalities and improve clinical outcomes in patients with KIRC.

Metabolic tumor reprogramming entails a series of adaptive mechanisms that support the high energy demands of rapidly growing and proliferating cancer cells [6]. Although specific metabolic changes, such as altered glycolysis, appear indeed necessary for malignant transformation, the deregulation of numerous metabolic pathways, including those linked to glutamine and lipid metabolism, has been closely associated with carcinogenesis and tumor progression [7–9]. Based on recognition of metabolic reprogramming as an essential hallmark of cancer, targeted approaches to redirect tumor metabolism have been developed and shown significant benefits in both preclinical and clinical studies [10, 11]. Paralleling these developments, investigators in the oncology field have increasingly applied integrated transcriptomics and metabolomics to identify metabolic biomarkers and their underlying molecular mechanisms. For example, Ma et al. uncovered key hepatocellular carcinoma (HCC) metabolism features and identified four significantly differential genes as promising biomarkers of patient survival [12]. In turn, a comprehensive molecular characterization of KIRC highlighted the critical role of metabolic alterations in kidney cancer progression [13]. Thus, efforts to identify effective tumor metabolism biomarkers may not only be of great significance to improve early diagnosis and prognosis, but to also help define novel therapeutic targets.

In the current study, we explored the prognostic significance of metabolism-related genes (MRGs) in KIRC patients through analysis of transcriptomics data obtained from The Cancer Genome Atlas (TCGA) database. We identified several MRGs differentially regulated in KIRC samples, and constructed after regression analyses a prognostic signature, composed of two MRGs, that was able to independently and accurately predict patient prognosis. Subsequently, a prognostic nomogram was established by integrating the prognostic signature and clinical variables. The metabolism-related prognostic signature identified in our study may help improve early KIRC diagnosis and stimulate new therapeutic strategies for KIRC.

## RESULTS

### Differentially expressed MRGs and functional enrichment analysis

**Figure 1:**
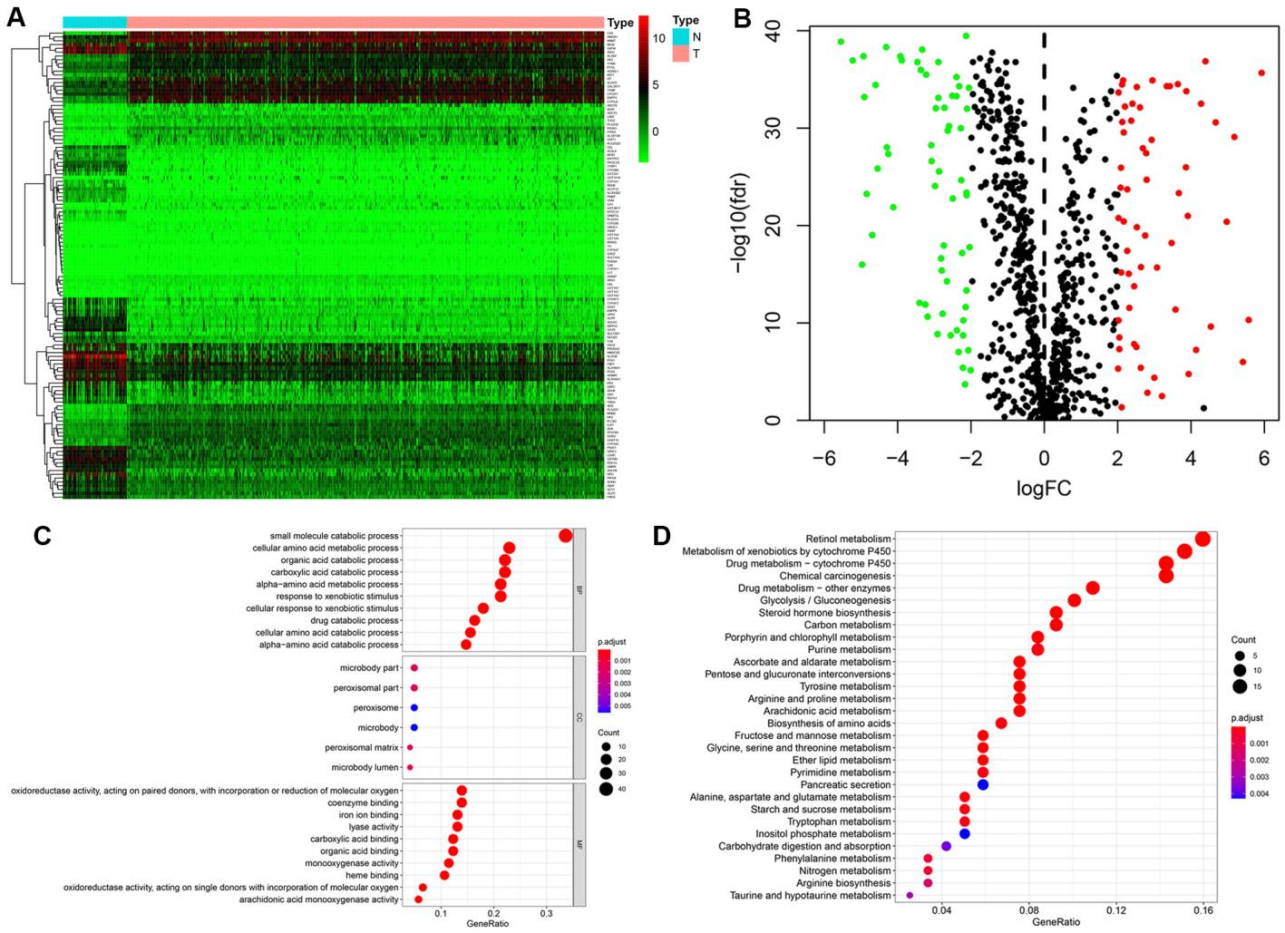
**Identification of differentially expressed metabolism-related genes (MRGs) and functional enrichment analysis.** (**A**) Heatmap of the 123 differently expressed MRGs between kidney cancer and normal tissues. (**B**) Volcano plot of the 123 differently expressed MRGs. Red and green dots indicate significantly upregulated and downregulated genes, respectively; black dots indicate similarly expressed genes. (**C**) GO analysis showing the enrichment of the differently expressed MRGs in biological process (BP), cellular component (CC), and molecular function (MF) terms. (**D**) KEGG pathway analysis for the differentially expressed MRGs.

The expression levels of 944 MRGs, obtained from The Molecular Signatures Database (MSigDB, were assessed in KIRC samples and normal kidney samples from the TCGA database using the Wilcoxon signed-rank test. A total of 123 differentially expressed MRGs, including 60 upregulated and 63 downregulated genes, were eventually identified based on the criteria of |log2FC| > 2 and FDR < 0.05 (Figure 1A, 1B).

Functional enrichment analysis was next performed to explore potential molecular mechanisms related to the differentially expressed MRGs. The most enriched GO terms in the biological process (BP) category were ‘small molecule catabolic process’, ‘cellular amino acid metabolic process’, and ‘organic acid catabolic process’. Significantly enriched GO terms related to the cellular component (CC) category included ‘microbody part’, ‘peroxisomal part’, and ‘peroxisomal matrix’. In the molecular function (MF) category, the differentially expressed MRGs were highly enriched in the terms ‘oxidoreductase activity’, ‘coenzyme binding’, and ‘iron ion binding’ (Figure 1C). In addition, KEGG pathway analysis revealed that these genes were notably associated with pathways in retinol metabolism, metabolism of xenobiotics by cytochrome P450, and drug metabolism − cytochrome P450 (Figure 1D).

### Identification of a metabolism-related prognostic signature for KIRC

**Figure 2:**
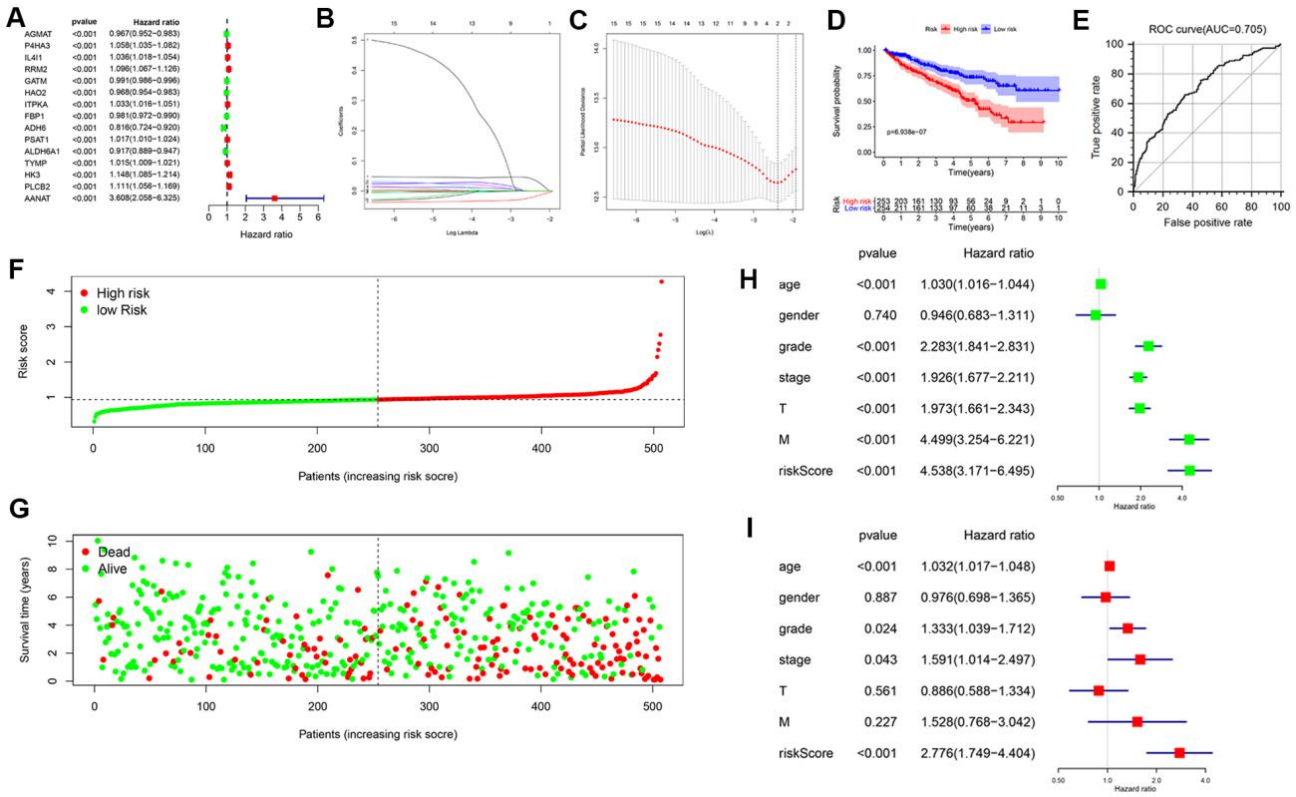
**Construction of a prognostic MRG signature based on TCGA-KIRC cohort.** (**A**) Identification of 15 MRGs in significant association with OS by univariate Cox regression analysis. (**B**, **C**) Screening of candidate MRGs used for the construction of the predictive signature using LASSO regression analysis. (**D**) Survival curves of KIRC patients assigned to high-and low-risk groups based on individual risk scores derived from the prognostic signature. (**E**) ROC analysis demonstrating survival prediction accuracy. (**F**) Distribution of risk scores. (**G**) Survival status for KIRC patients in the high- and low-risk groups. Univariate (**H**, **I**) multivariate Cox regression analysis were used to verify that the metabolism-related prognostic signature represents an independent prognostic factor for KIRC patients.

To explore the prognostic value of MRGs in renal cancer progression, we performed univariate Cox regression analysis to examine potential relationships between the expression levels of the 123 MRGs and patient OS. Results demonstrated that 15 MRGs were significantly associated with OS (P < 0.01) (Figure 2A). Among those, P4HA3, IL4I1, RRM2, ITPKA, PSAT1, TYMP, HK3, PLCB2, and AANAT were considered as risk genes (HR > 1), while AGMAT, GATM, HAO2, FBP1, ADH6, and ALDH6A1 were considered as protective genes (HR < 1). We then used least absolute shrinkage and selection operator (LASSO) Cox regression on the above-mentioned 15 MRGs to identify the most optimal risk score model for predicting survival in KIRC patients (Figure 2B, 2C). Eventually, RRM2 and ALDH6A1 were retained as target genes, and their respective coefficients were calculated to construct the metabolism-related prognostic signature.

We constructed the OS prognostic signature based on the expression of the 2 target genes and their prognostic coefficients using the following formula: Risk score= (0.0361 × expression level of RRM2) + (–0.0184 × expression level of ALDH6A1). According to the median risk score, 253 and 254 KIRC patients were sorted into a high-risk group and a low-risk group, respectively. The Kaplan-Meier curve displayed a significant difference in OS between the high- and the low-risk groups (5-year survival rate, 49.3% vs. 72.6%, respectively; P <0.001) (Figure 2D). We then applied the ROC curve to evaluate the predictive accuracy of the signature. The area under the ROC curve was 0.705, suggesting a moderate prognostic value (Figure 2E). In addition, the distributions of risk scores and survival status of patients were ranked according to the risk scores (Figure 2F, 2G).

### Evaluation of the prognostic signature as an independent prognostic factor

We performed univariate and multivariate Cox regression analyses to further determine whether the prognostic signature could serve as an independent prognostic factor. Univariate analysis revealed that age, grade, AJCC stage, T stage, M stage, and risk score were significantly associated with OS (Figure 2H). Subsequent results showed that age (P < 0.001), grade (P = 0.024), AJCC stage (P = 0.043), and risk score (P < 0.001) were still significantly correlated with OS in multivariate analyses (Figure 2I). These data indicate that our metabolism-related prognostic signature is an independent prognostic factor for KIRC patients.

### Clinical utility of the prognostic signature

**Figure 3:**
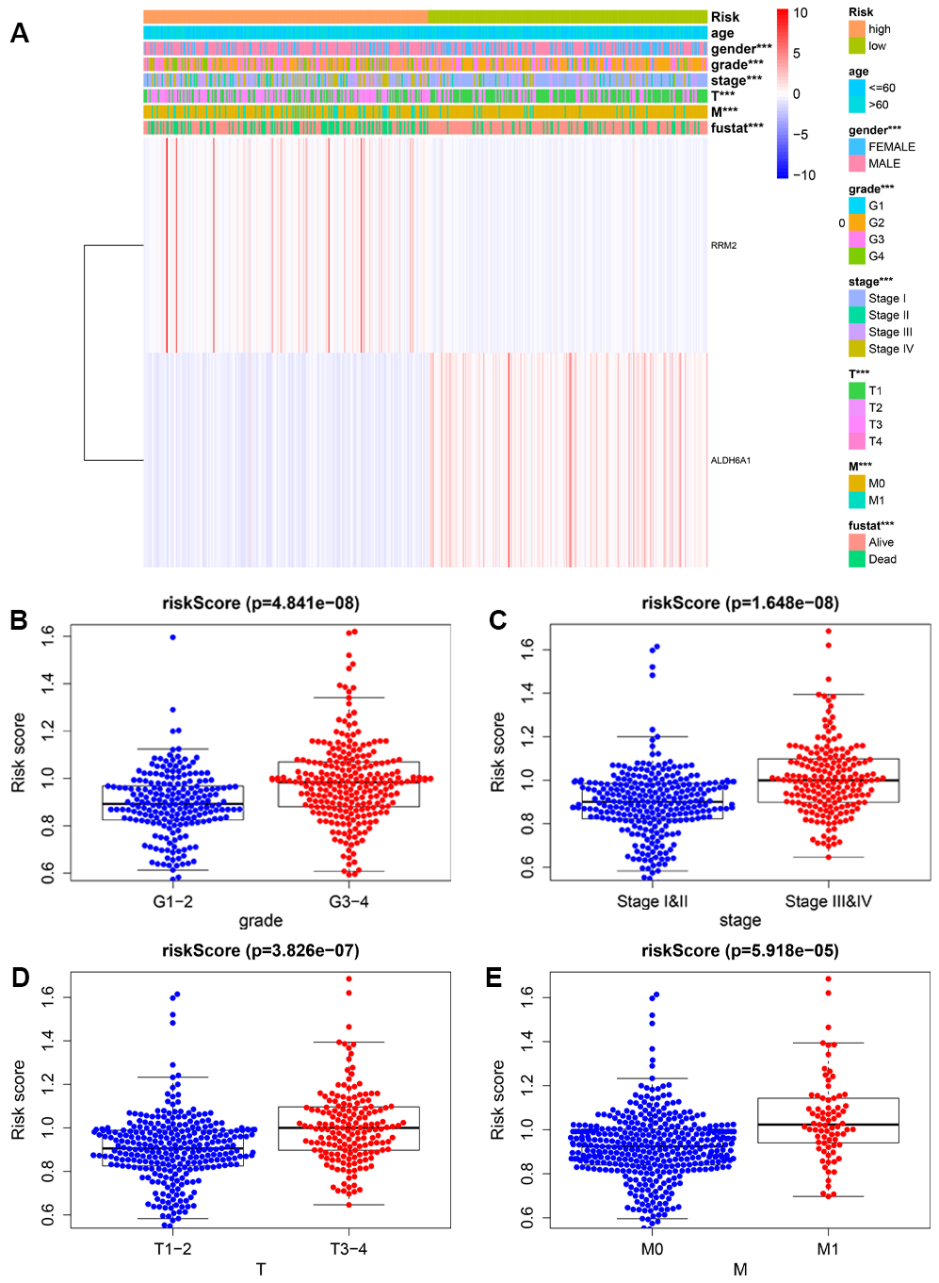
**Relationship between risk scores and clinicopathological features.** (**A**) Heatmap showing the distribution of clinical parameters and the expression of the two signature genes between the low- and high-risk groups. *** *P* < 0.001. The plots below show the association of risk score with grade (**B**), AJCC stage (**C**), T stage (**D**), and M stage (**E**).

We further explored the relationship between the metabolism-related prognostic signature and various clinical parameters. The expression levels of the two signature MRGs in the high- and low-risk groups are shown in a heatmap (Figure 3A). This analysis showed that RRM2 and ALDH6A1 were expressed at high and low levels, respectively, in the high-risk group. Additionally, we observed significant differences between low- and high-risk groups in grade (*P* = 4.841e−08), AJCC stage (*P* = 1.648e−08), T stage (*P* = 3.826e−07), and M stage (*P* = 5.918e−05) (Figure 3B–3E).

**Figure 4:**
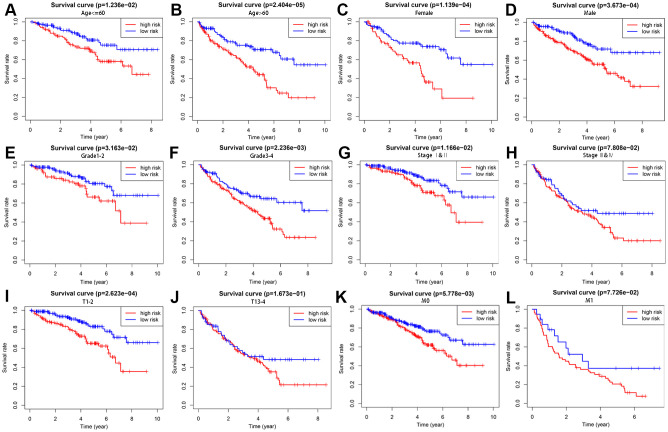
**Survival analysis of high- and low-risk groups stratified by clinical parameters.** Differences in OS between high- and low-risk groups stratified by age (**A**, **B**), gender (**C**, **D**), grade (**E**, **F**), AJCC stage (**G**, **H**), T stage (**I**, **J**), and N stage (**K**, **L**).

To better evaluate the survival outcomes and define the broad applicability of the prognostic signature, we next performed survival analyses stratified by age, gender, tumor grade, AJCC stage, T stage, and M stage. As shown in Figure 4, patients in the high-risk group had significantly shorter OS than those in the low-risk group for cases with age ≤ 60 (*P* = 1.236e−02), age > 60 (*P* = 2.404e−05), female gender (*P* = 1.139e−04), male gender (*P* =3.673e−04), G1-2 (*P* = 3.163e−02), G3-4 (*P* = 2.236e−02), AJCC stage I and II (*P* =1.166e−02), T1-2 (*P* = 2.623e−04), and M0 (*P* = 5.778e−03). In contrast, no significant differences were observed for OS between high- and low-risk groups for KIRC patients with AJCC stage III and IV (*P* = 7.808e−02), T3-4 (*P* = 1.673e−01), and M1 (*P* = 7.726e−02).

**Figure 5:**
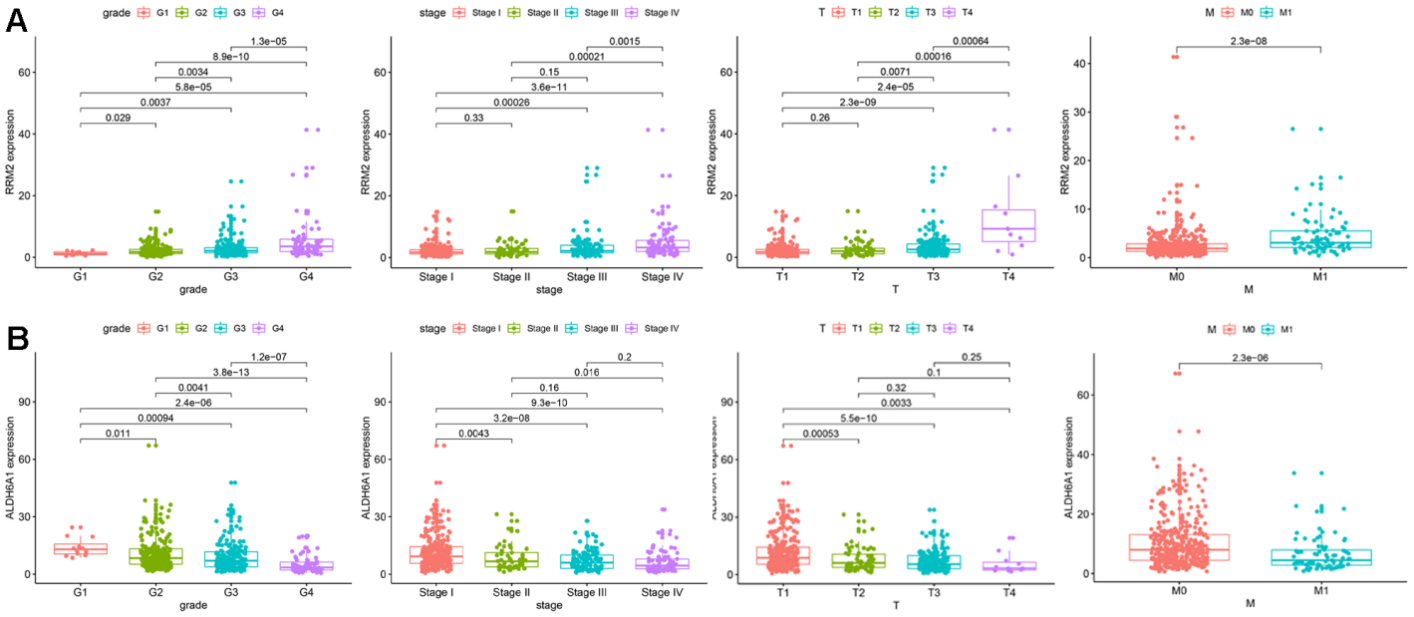
**Correlation between the expression of each signature gene and clinical parameters.** (**A**) RRM2. (**B**) ALDH6A1.

We then analyzed the relationship between the expression level of each target gene in the prognostic signature and clinicopathological features to assess the function of the two genes in disease progression. The results indicated that enhanced RRM2 expression was significantly associated with advanced tumor stage and high-grade tumor, suggesting in turn a positive correlation between RRM2 expression and poor prognosis in KIRC. Indeed, the highest RRM2 expression was detected in the most progressive clinicopathological stage, that is, G4 and stage IV, T4, and M1 (Figure 5A). In contrast, ALDH6A1 expression tended to gradually decrease with KIRC progression (Figure 5B), which suggested that ALDH6A1 is a protective factor for KIRC.

### Construction and validation of a predictive nomogram

**Figure 6:**
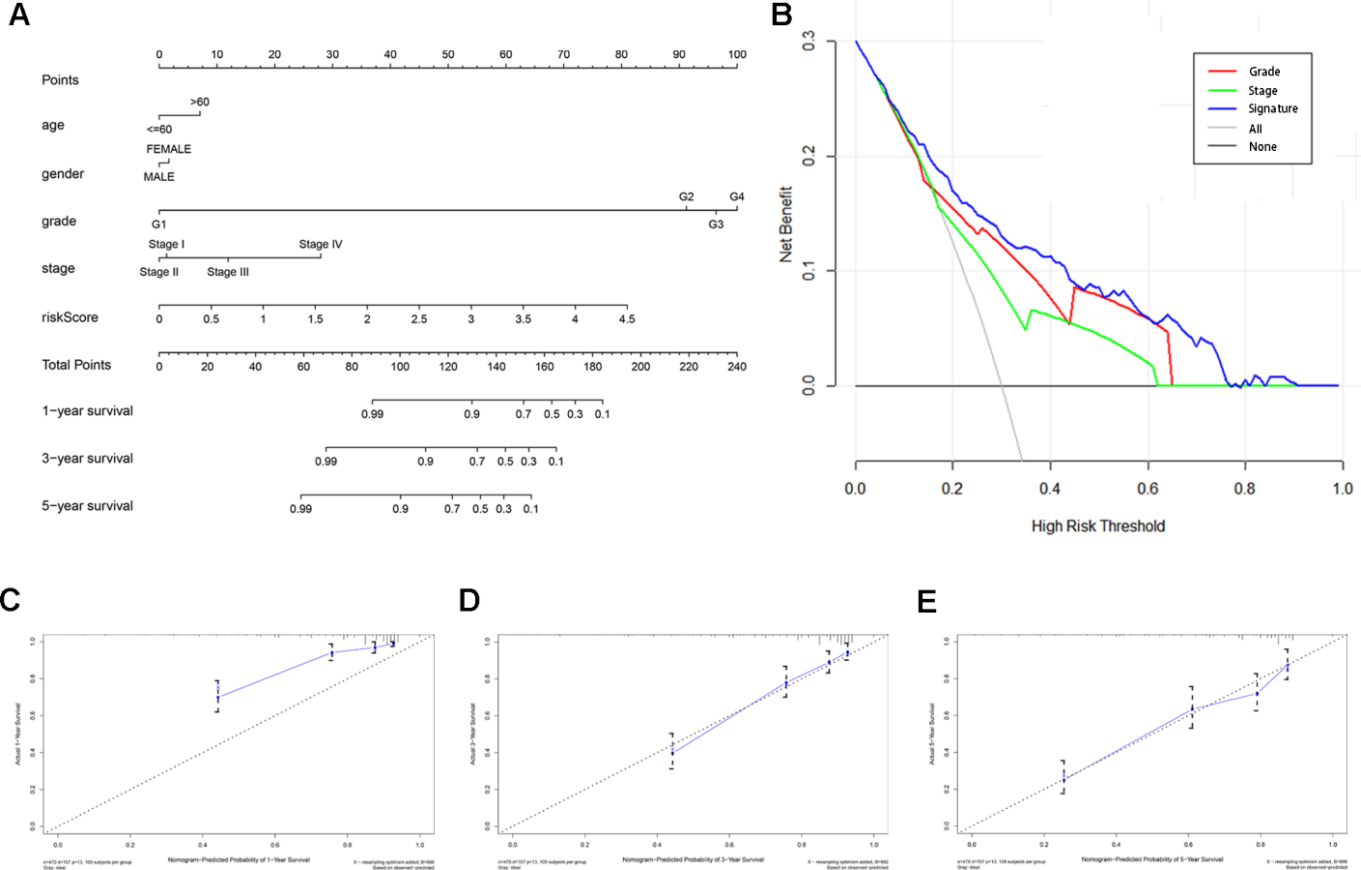
**Construction and validation of a predictive nomogram.** (**A**) Nomogram for predicting 1-, 3-, and 5-year OS of KIRC patients in the TCGA cohort. (**B**) DCA showing that the nomogram confers higher net benefit to predict OS when the threshold probability is larger than 3%. (**C**–**E**) Calibration curves indicating the performance of the nomogram in predicting 1-, 3- and 5-year OS compared to an ideal model.

A predictive nomogram was constructed by incorporating the prognostic signature and several clinical parameters to generate individual numerical probabilities of OS (Figure 6A). The C-index of the developed nomogram was 0.774. DCA demonstrated that the nomogram provided a higher net benefit in predicting OS if the threshold probability was larger than 3% (Figure 6B). Additionally, the nomogram displayed an obviously higher net benefit than did tumor grade and AJCC stage. The calibration curves indicated that the nomogram performed well in predicting 1-, 3- and 5-year OS compared with the ideal model (Figure 6C–6E). These results are thus indicative of the reliability and predictability of the nomogram.

### External validation of the expression and prognostic value of RRM2 and ALDH6A1

**Figure 7:**
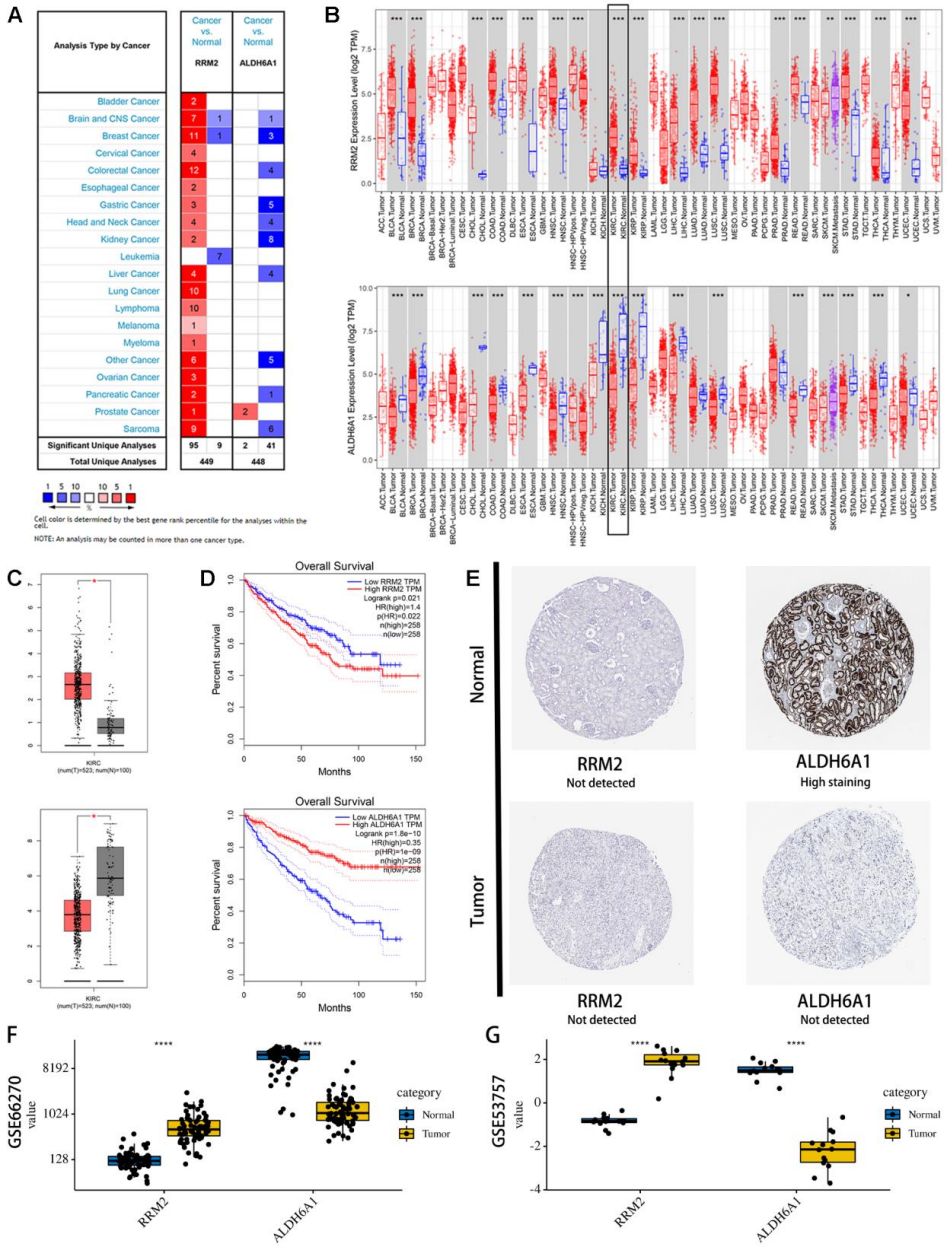
**Expression levels and prognostic value of the signature genes.** (**A**) Expression profiles of RRM2 and ALDH6A1 transcripts in the Oncomine database. (**B**) Expression profiles of RRM2 and ALDH6A1 transcripts in various cancers on the TIMER database. (**C**) Expression profiles of RRM2 and ALDH6A1 transcripts in the GEPIA database. (**D**) Univariate survival analysis (Kaplan-Meier curves) for RRM2 and ALDH6A1. (**E**) Immunohistochemistry images of RRM2 and ALDH6A1 expression in KIRC and normal kidney tissues. Examples were retrieved from the Human Protein Atlas database. (**F**) Verification of RM2 and ALDH6A1 expression in KIRC and normal tissues in the GSE53757 database. (**G**) Verification of RM2 and ALDH6A1 expression in KIRC and normal tissues in the GSE66270 database.

Consistent with our results, analysis of the Oncomine (Figure 7A), TIMER (Figure 7B), and GEPIA (Figure 7C) databases revealed that RRM2 expression was significantly upregulated, while ALDH6A1 expression was significantly downregulated, in KIRC samples compared with normal ones. Moreover, similar expression trends for these two genes were frequently detected in various cancer types (Figure 7B). In addition, the prognostic value of each gene was further confirmed by Kaplan Meier analysis in the GEPIA database, which indicated favorable prognosis for the RRM2 low-expression and the ALDH6A1 high-expression groups (Figure 7D).

To determine protein expression levels of RRM2 and ALDH6A1, we interrogated the HPA database. The information available indicated that ALDH6A1 expression in kidney cancer is significantly lower than in normal kidney tissue (Figure 7E). In contrast, no significant difference was found for RRM2 expression between kidney cancer and normal tissues. In parallel, gene expression levels of RRM2 and ALDH6A1 were further verified in two independent cohorts (GSE53757, Figure 7F, and GSE66270, Figure 7G) in the GEO database. Results showed that RRM2 was significantly overexpressed, while ALDH6A1 was significantly underexpressed, in KIRC samples compared with normal ones. Therefore, our assessment of multiple datasets was highly consistent, especially at the transcriptional level, with our expression data for the two prognostic signature genes.

### GSEA analysis of signature genes

**Figure 8:**
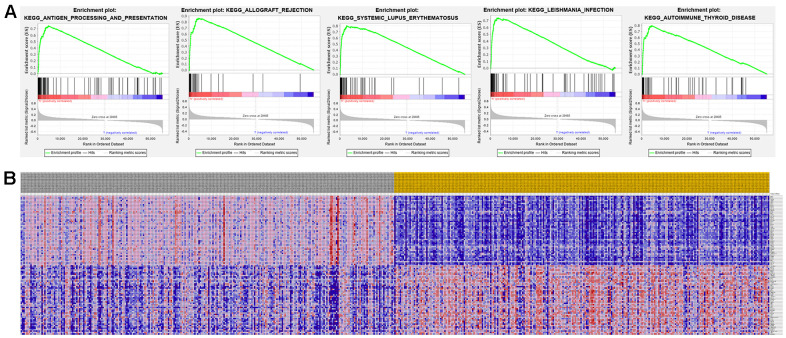
**Significant RRM2-related genes and corresponding hallmark pathways in KIRC samples identified via GSEA.** (**A**) Enrichment plots for the most significant pathways involving RRM2-related genes. (**B**) Heatmap showing transcriptional expression profiles of the top 50 genes for each phenotype.

**Figure 9:**
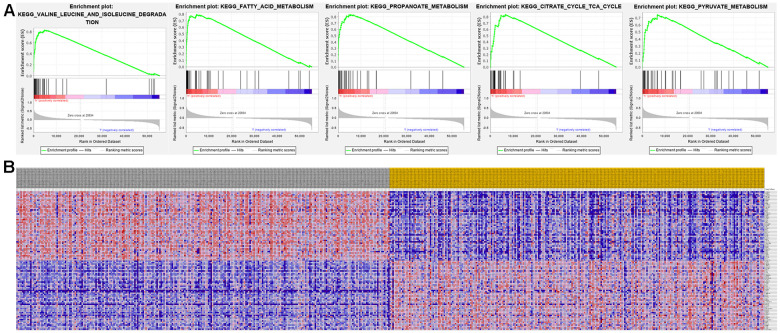
**Significant ALDH6A1-related genes and corresponding hallmark pathways in KIRC patients identified via GSEA.** (**A**) Enrichment plots for the most significant pathways involving ALDH6A1-related genes. (**B**) Heatmap showing transcriptional expression profiles of the top 50 genes for each phenotype.

The top 50 genes with significant correlation with our two prognostic genes were retrieved using GSEA. Subsequently, we performed MSigDB Hallmark analysis for RRM2 and ALDH6A1. Results indicated that the most significant pathways related with RRM2 included ANTIGEN PROCESSING AND PRESENTATION, ALLOGRAFT REJECTION, SYSTEMIC LUPUS ERYTHEMATOSUS, LEISHMANIA INFECTION, and AUTOIMMUNE THYROID DISEASE (Figure 8A). A heatmap showing transcriptional expression profiles of the top 50 RRM2-correlated genes for each phenotype (Figure 8B). Correspondingly, GSEA showed that significantly enriched pathways for ALDH6A1 included VALINE LEUCINE AND ISOLEUCINE DEGRADATION, FATTY ACID METABOLISM, PROPANOATE METABOLISM, CITRATE CYCLE TCA CYCLE, and PYRUVATE METABOLISM (Figure 9A). Transcriptional expression profiles of the top 50 genes associated with ALDH6A1 for each phenotype are shown in a heatmap on Figure 9B.

## DISCUSSION

Dysregulation of tumor cell metabolism represents a hallmark of cancer [14, 15]. Early evidence indicated that inactivation of metabolic genes with tumor suppressor properties mediates a direct genetic link to altered tumor metabolism [16]. In turn, further research unveiled a large number of mutations in oncogenes and tumor suppressor genes that drive distinct cell proliferation and survival programs in tumor cells by effecting metabolic reprogramming [17]. In common with most cancers, KIRC is a disease affected by complex gene interactions determining dysregulated cellular metabolism [18]. Recently, several characteristic kidney cancer-related genes, including VHL, MET, FLCN, TSC1, TSC2, FH, and SDH, have been reported to affect the metabolic stress response [19]. Sunitinib, a tyrosine kinase inhibitor that targets the VHL pathway, is currently the most commonly used drug to treat advanced and metastatic RCC. However, the partial response rate for sunitinib in advanced KIRC patients is only 31% [20]. Therefore, identifying a reliable metabolic signature predictive of tumor progression will not only assist in understanding the molecular mechanisms involved, but might also provide KIRC patients with more effective targeted treatments.

In this study, we investigated the relationships between the expression profiles of MRGs, retrieved from the MSigDB, and the prognosis of KIRC patients by analysis of transcriptomic data. This approach represents a valuable paradigm in cancer biology to ensure the reliability of metabolic biomarkers. We first examined differences in MRG expression between KIRC tissues and normal tissues from TCGA and detected 60 upregulated and 63 downregulated MRGs in KIRC patients. GO and KEGG analyses of the differentially expressed MRGs confirmed that those genes were highly enriched in metabolic pathways. On KEGG pathway analysis, the most significant pathway was retinol metabolism, which is consistent with the major role of retinol and related metabolites in the growth and differentiation of normal and malignant cells, including KIRC [21, 22]. Based on univariate Cox-LASSO regression analyses, we then constructed a metabolism-related signature consisting of two prognostic MRGs, i.e. RRM2 and ALDH6A1. ROC-AUC estimations indicated good performance of the prognostic signature. A subsequent clinical application analysis further demonstrated that the signature could accurately discriminate prognostic outcomes between high- and low-risk patient groups.

Our study further demonstrated that the prognostic signature was an independent prognostic factor for OS in KIRC patients, suggesting a strong prognostic potential related to the tumor’s metabolic status. We also found that the two-gene signature-based risk score for each patient was an important clinical variable in the derived nomogram, which indicated that the signature was highly accurate in predicting KIRC outcomes. The results from calibration plots and the C-index showed that the generated nomogram performed well in terms of discriminating clinical outcomes in KIRC patients, while DCA demonstrated for the nomogram a higher net prognostic benefit than that provided by tumor grade and AJCC stage.

The RRM2 protein is a subunit of the ribonucleotide reductase (RNR), an enzyme that catalyzes the de novo synthesis of deoxyribonucleoside diphosphates (dNDPs) to provide dNTP precursors for DNA synthesis. Cancer cells rely on extensive dNTP supply to sustain continuous growth; overexpression of RRM2 is closely related to tumorigenesis and disease progression in many cancer types, leading to its recognition as an effective target of anticancer therapies [23, 24]. For example, in *vitro* and *vivo* experiments showed that overexpression of RRM2 promoted epithelial-mesenchymal transition, whereas knockdown of RRM2 inhibited its oncogenic function in prostate cancer [25]. Meanwhile, Sun et al. found that RRM2 was a positive regulator in the progression of glioma, promoting glioma cell proliferation and migration via ERK1/2 and AKT signaling [26].

ALDH6A1, a mitochondrial methylmalonate semialdehyde dehydrogenase, is involved in lipid metabolism and in the catabolic breakdown of valine and thymine [27, 28]. ALDH6A1 expression was found to be markedly downregulated in KIRC tissues, in association with poor survival. Accordingly, its overexpression significantly decreased cell proliferation and migration and impaired oncologic metabolism in KIRC cells [29]. Results from another study employing extensive quantitative proteomic profiling analysis and molecular characterization suggested that hepatic neoplastic transformation inhibits the expression of ALDH6A1. Based on clinical expression data, ALDH6A1 was proposed as a potential molecular signature for HCC [30]. Meanwhile, two-dimensional gel electrophoresis and mass spectrometry analyses indicated that ALDH6A1 was highly specific to metastatic tumor cells and its expression was significantly reduced in metastatic prostate cancer [31]. Indeed, in light of the close affiliation of dysregulated ALDH isozymes with cancer stem cell growth, ALDHs have gained relevance as novel biomarkers and potential therapeutic targets in cancer research [32].

Our study presents some limitations. First, this study was a retrospective data collection and analysis, subjected therefore to inevitable selection and information bias. Second, as the expression levels of the two genes were verified only in public databases, proving the prognostic value of this signature in independent cohorts is warranted to expand its applicability. Third, it is necessary to perform further experimental verification in *vivo* and *vitro* to illustrate the mechanisms underlying the regulation of the predictive metabolic genes.

In summary, by cross-referencing a large MRG dataset with transcriptomic data from the TCGA-KIRC cohort, we extracted two metabolism-related genes and constructed a novel metabolic signature with the ability to accurately and independently predict the prognosis of KIRC patients. Furthermore, the gene signature-based risk score for each patient proved to be an important clinical variable in our proposed nomogram, which could predict with high confidence 3- and 5-year survival probabilities for individual KIRC patients. These findings indicate that the metabolism-related gene signature identified herein might be clinically significant, aiding in KIRC patient prognosis and helping design novel cancer metabolism-targeted therapies.

## MATERIALS AND METHODS

### Study cohort

RNA sequencing transcriptome data and clinical information were downloaded for 539 KIRC and 72 normal tissues from The Cancer Genome Atlas (https://cancergenome.nih.gov/). The extracted data were normalized and processed by *log2* transformation. KIRC samples with incomplete data or corresponding to survival times shorter than 30 days were excluded. Metabolism-related gene (MRG) sets were obtained from the Kyoto Encyclopedia of Genes and Genomes (KEGG) pathways in The Molecular Signatures Database (MSigDB, https://www.gsea-msigdb.org/gsea/msigdb/) [33]. A total of 944 genes were selected for further analysis after removing duplicates.

### Identification of differentially expressed MRGs and enrichment analysis

Differentially expressed MRGs between cancer samples and normal control samples were identified by LIMMA package in R language [34] with the following thresholds: |log2 fold change (FC)| > 2.0 and a false discovery rate (FDR) corrected p-value < 0.05 (calculated by the Benjamini and Hochberg procedure). A series of functional enrichment analyses was performed to explore potential roles for the differentially expressed MRGs using gene ontology (GO) and KEGG. The ggplot2 and enrichplot R packages were used to supply the visual enrichment maps of annotation analysis results to help interpretation. P < 0.05 was regarded as statistically significant.

### Construction and evaluation of the prognostic signature

The relationship between the expression levels of the differentially expressed MRGs and OS was first examined via univariate Cox regression analysis. Genes with significant prognostic value (*P* < 0.05) were included for subsequent validation. To obtain the most optimal MRGs and to control the complexity of the model, we carried out a LASSO Cox regression analysis with the glmnet package. LASSO Cox regression is a robust model building method that reduces the dimension of the model and prevents data overfitting [35]. After that, several target genes were obtained to develop the prognostic signature. Based on the signature, the risk score for each patient was calculated using the following formula:$\text{Risk}\;\text{Score}=\sum_{i=1}^{n}Coef(i)\times x(i),$ where *n* represents the number of module genes, *Coef*(*i*) denotes the estimated regression coefficient by LASSO analysis, and *x*(*i*) indicates the relative expression level of each MRG.

The median risk score was taken as the cut-off point to separate all KIRC patients into high-risk and low-risk groups. The Kaplan–Meier method and the log-rank test were used to compare the difference in OS between high- and low-risk groups. Time-dependent receiver operating characteristic (ROC) curve analysis was conducted to assess the predictive accuracy of the prognostic signature; the value of the area under the ROC curve (AUC) ranged from 0.5 (no predictability) to 1 (perfect predictability).

Univariate and multivariate Cox regression analyses were applied to assess whether the metabolism-related signature could be an independent predictor of OS for the TCGA-KIRC cohort. Age, gender, tumor grade, American Joint Committee on Cancer (AJCC) stage, T stage, and M stage were used as covariates. N stage was not analyzed because of a large proportion of missing data.

To detect the prognostic value of the risk score in different subgroups, stratified survival analysis was carried out according to clinical characteristics related to the prognosis: age (≤ 65 and > 65 years), gender, tumor grade (G1-2 and G3-4), AJCC stage (I/II and III/IV), T stage (T1-2 and T3-4), and M (M0 and M1) stage. The relationship between the expression of each target gene and clinical parameters was also compared to further understand the impact of each individual target gene in our prognostic signature.

### Development of a predictive nomogram

A nomogram incorporating several clinical variables (age, gender, grade, AJCC stage, T stage, and M stage) and the risk score calculated from the prognostic signature was developed to evaluate the probability of 1-, 3-, and 5-year OS for TCGA-KIRC patients. Subsequently, the discriminative and predictive abilities of the nomogram were evaluated by determining the concordance index (C-index) and by generating calibration plots. C-index values range from 0.5 to 1.0, indicating respectively no discriminating power and perfect discriminating ability. A reference line with a slope of one in the calibration plots represents perfect calibration. Decision curve analysis (DCA) was performed to evaluate the clinical utility of the signature-based nomogram model by quantifying the net benefits under different threshold values.

### External verification of the prognostic genes

To further evaluate the reliability of the prognostic gene signature set, we investigated the expression of RRM2 and ALDH6A1 in both tumor and normal tissues using Oncomine (https://www.oncomine.org/resource/main.html) and Tumor Immune Estimation Resource (TIMER, https://cistrome.shinyapps.io/timer/) databases. In addition, the Gene Expression Profiling Interactive Analysis (GEPIA, http://gepia.cancer-pku.cn/) online tool was accessed to verify the prognostic value of the above target genes through survival analysis. The expression of the prognostic genes in the gene signature was further investigated at the protein level using the Human Protein Atlas (HPA, https://www.proteinatlas.org/) database. Then, differences in the expression of these target genes between tumor and normal tissue samples were verified in the Gene Expression Omnibus (GEO) database (https://www.ncbi.nlm.nih.gov/geo/).

### Gene set enrichment analysis (GSEA)

GSEA (http://www.broadinstitute.org/gsea) was performed using 1000 permutations for each task to identify significantly enriched pathways. The minimum and maximum criteria for selection of gene sets from the collection were 15 and 500 genes, respectively. Significantly related genes were defined with a nominal *P* value < 0.05 and FDR < 0.25.

### Statistical analyses

All statistical analyses were performed with R 3.6.2 (https://www.r-project.org/). All analyses were two-sided and statistical significance was defined as *P* < 0.05.
